# Shared molecular mechanisms between colorectal cancer and cardiovascular disease drive perioperative neurological vulnerability

**DOI:** 10.3389/fgene.2026.1902646

**Published:** 2026-09-22

**Authors:** Ling Jiang, Mingqian Yang, Yan Yang, Li Yang, Liyang Xu

**Affiliations:** 1 Department of Anesthesiology, Chongqing Jiangjin Central Hospital, Chongqing, China; 2 Department of Anesthesiology Surgery, Ezhou Central Hospital, Ezhou, China; 3 Department of Anesthesiology Surgery, Zibo Central Hospital, Zibo, China; 4 Department of Otolaryngology Head and Neck Surgery, Chongqing Jiangjin Central Hospital, Chongqing, China; 5 Department of Anesthesiology, Huai’an Hospital of Huai’an City (Huai’an Clinical Medical College of Jiangsu University), Huai’an, China

**Keywords:** cardio-oncology, cardiovascular disease, colorectal cancer, frailty, immune dysregulation, metabolic reprogramming, multi-omics, neuroinflammation

## Abstract

**Background:**

Colorectal cancer (CRC) and cardiovascular disease (CVD) share overlapping molecular pathogenic axes—including chronic systemic inflammation, immune dysregulation, metabolic reprogramming, and cellular senescence—that collectively amplify perioperative neurological vulnerability in elderly patients. Postoperative delirium (POD), the most prevalent acute neuropsychiatric complication following major oncological surgery, emerges at the intersection of these shared pathogenic pathways: tumor-driven neuroinflammation, anesthesia-induced cerebral hypoperfusion, cardio-metabolic derangements, and frailty-associated multi-system reserve failure collectively prime the aging brain for acute cognitive disruption. Despite this mechanistic convergence, clinically actionable risk stratification tools anchored in this cardio-oncological molecular framework remain absent.

**Objective:**

To construct and validate a precision nomogram for POD prediction in elderly CRC patients by integrating preoperative frailty phenotyping, cardio-metabolic biomarkers, and anesthesia-related intraoperative indicators, positioning each predictor within the shared CRC-CVD molecular landscape and translational omics evidence.

**Methods:**

A retrospective cohort of 283 elderly patients (≥65 years) undergoing elective radical resection for CRC (January 2021–June 2024) was analyzed. Preoperative frailty was assessed using the Fried Frailty Phenotype across five physiological dimensions. POD was diagnosed by the Confusion Assessment Method (CAM). Data encompassed demographic characteristics, cardio-metabolic comorbidities, inflammatory and nutritional biomarkers, frailty dimensions, and anesthesia-related indicators. Multivariate logistic regression identified independent predictors, and a nomogram was constructed using the rms package (R 4.3.0). Model performance was evaluated by C-index, AUC, Hosmer–Lemeshow test, calibration curves, and decision curve analysis (DCA), with Bootstrap internal validation (1,000 iterations). Each predictor is mechanistically contextualized within existing multi-omics, proteomics, and translational CRC-CVD molecular biology evidence.

**Results:**

POD developed in 53/283 patients (18.7%). Seven independent predictors were identified: advanced age (OR = 1.125, 95%CI: 1.058–1.196), low education level (OR = 2.341, 95%CI: 1.287–4.259), frailty score ≥3 items (OR = 4.862, 95%CI: 2.154–10.975), intraoperative hypotension (OR = 3.524, 95%CI: 1.723–7.209), intraoperative hypothermia (OR = 3.187, 95%CI: 1.542–6.589), anesthesia duration ≥200 min (OR = 2.678, 95%CI: 1.328–5.400), and albumin <35 g/L (OR = 2.814, 95%CI: 1.245–6.358). The nomogram achieved C-index 0.842 (95%CI: 0.792–0.892), Bootstrap-corrected C-index 0.835, AUC 0.842 (95%CI: 0.791–0.893), sensitivity 79.2%, specificity 82.6%, and NPV 92.7%. DCA demonstrated robust net clinical benefit across threshold probabilities of 0.10–0.70.

**Conclusion:**

This nomogram operationalizes the convergent CRC-CVD molecular pathogenic landscape into a clinically deployable perioperative precision medicine tool. Its predictors—spanning neuroinflammatory priming, vascular homeostatic failure, metabolic-nutritional derangement, and phenotypic frailty—map directly onto shared molecular axes of CRC and CVD, validating the cardio-oncological framework as a foundation for POD risk stratification. Future multi-omics integration—incorporating inflammatory proteomics, metabolomic signatures, and machine-learning models—will further advance individualized perioperative management in elderly CRC patients.

## 1 Introduction

### 1.1 The cardio-oncological convergence: CRC and CVD as a shared disease continuum

Colorectal cancer (CRC) and cardiovascular disease (CVD) are the two dominant causes of global morbidity and mortality, and emerging evidence compels their recognition not as independent pathological entities but as components of a shared disease continuum. Large-scale epidemiological data demonstrate a bidirectional relationship: CRC patients carry a substantially elevated risk of cardiovascular events driven by tumor-derived systemic inflammation, metabolic dysregulation, pro-coagulant states, and treatment-associated cardiotoxicity, while CVD patients exhibit elevated long-term cancer incidence mediated by shared upstream pathogenic drivers including chronic inflammation, clonal hematopoiesis of indeterminate potential (CHIP), immune senescence, and vascular niche remodeling. These observations establish CRC and CVD as mechanistic co-travelers across epidemiological, molecular, and clinical dimensions.

At the molecular level, CRC and CVD converge on a core set of pathogenic pathways: NF-κB-driven chronic inflammation with sustained elevation of interleukin-6 (IL-6), tumor necrosis factor-alpha (TNF-α), and C-reactive protein (CRP); metabolic reprogramming characterized by altered glucose and lipid metabolism, mitochondrial dysfunction, and reactive oxygen species (ROS) accumulation; immune dysregulation involving macrophage polarization, T Cell exhaustion, and endothelial activation; and dysregulated cell-death cascades including apoptosis resistance, pyroptosis, and ferroptosis. These convergent molecular axes are not merely biological curiosities—they constitute the mechanistic substrate through which CRC pathobiology amplifies cardiovascular risk and, critically, through which both disease trajectories intersect with perioperative neurological vulnerability.

### 1.2 Postoperative delirium as a cardio-oncological neurological phenotype

Postoperative delirium (POD) is an acute neuropsychiatric syndrome characterized by impaired consciousness, inattention, and fluctuating cognitive dysfunction, representing the most prevalent and consequential neurological complication in elderly cancer surgery patients. Reported incidence rates reach 15%–25% in elderly abdominal surgery cohorts (Hu et al., 2024), with consequences extending far beyond the acute episode: accelerated cognitive decline, increased dementia risk, prolonged ICU and hospital stay, elevated long-term mortality, and substantially reduced quality of life (Lin et al., 2022; Mohanty et al., 2022). Yet POD remains poorly integrated into CRC-CVD molecular frameworks.

This integration is biologically imperative. POD in elderly CRC patients arises precisely at the intersection of shared CRC-CVD molecular pathways: the tumor-driven chronic inflammatory milieu (elevated IL-6, TNF-α, CRP) primes cerebral microglia for hyperactivation; cancer-associated endothelial dysfunction and vascular remodeling compromise cerebral autoregulation and blood-brain barrier (BBB) integrity; cardiovascular comorbidities—endemic in this population—impose additional hemodynamic and perfusion constraints; metabolic reprogramming in CRC depletes substrates required for neural repair; and the frailty phenotype—increasingly recognized as the clinical readout of converged CRC-CVD molecular aging—amplifies multi-system vulnerability to perioperative stressors. Viewed through this lens, POD is not merely a perioperative complication but a neurological manifestation of the shared CRC-CVD pathogenic landscape under the acute stress of surgery and anesthesia.

### 1.3 Multi-omics perspectives on CRC-CVD-POD intersection

The advent of multi-omics technologies—transcriptomics, proteomics, metabolomics, and epigenomics—has begun to map the molecular architecture of CRC-CVD convergence with unprecedented resolution. Transcriptomic profiling of CRC tumors and atherosclerotic plaques reveals shared upregulation of inflammatory gene networks (NF-κB, STAT3 pathways), macrophage activation signatures, and angiogenic programs. Proteomic analyses identify circulating biomarkers—including SASP-associated proteins, endothelial injury markers, and coagulation factors—that simultaneously reflect tumor progression, cardiovascular disease activity, and systemic biological aging. Metabolomic signatures of CRC and CVD overlap in dysregulated lipid metabolism, impaired mitochondrial energy production, and altered amino acid profiles—derangements that directly compromise neural substrate availability. Epigenomic studies reveal shared DNA methylation patterns, particularly at aging-associated CpG loci, linking CHIP-driven clonal hematopoiesis to both atherogenesis and tumor immune evasion.

Critically, these same molecular signatures intersect with neurological vulnerability. Systemic inflammation primes the neuroinflammatory axis; vascular and metabolic derangements impair cerebral homeostasis; immune dysregulation compromises neuroprotection. The emerging field of cardio-oncological neuroscience thus identifies shared molecular drivers as upstream determinants of POD risk—yet validated clinical tools incorporating this framework for perioperative risk stratification remain absent.

### 1.4 Study rationale and objectives

This study addresses this critical gap by constructing and validating a precision nomogram for POD prediction in elderly CRC patients (Hu et al., 2024; Song et al., 2024). The model integrates preoperative frailty phenotyping—which captures the clinical phenotype of converged CRC-CVD molecular aging—with cardio-metabolic biomarkers and anesthesia-related intraoperative indicators that reflect the acute hemodynamic, thermal, and pharmacological perturbations superimposed on this chronic molecular vulnerability. Each predictor is mechanistically positioned within the shared CRC-CVD molecular landscape and contextualized by translational multi-omics evidence. This work directly addresses the Research Topic’s scope: translational applications of omics data to guide perioperative management and surgical outcomes in cancer patients, with particular emphasis on the molecular intersections between CRC and cardiovascular pathobiology.

## 2 Shared molecular pathogenic axes between CRC and CVD: relevance to perioperative neurological vulnerability

### 2.1 Chronic systemic inflammation as the central convergence point

Chronic low-grade inflammation represents the most extensively characterized shared molecular axis between CRC and CVD. In CRC, NF-κB activation—driven by tumor-derived cytokines, microbiome dysbiosis, and tumor microenvironment (TME) remodeling—sustains elevated circulating IL-6, TNF-α, IL-1β, and CRP. These mediators promote tumor invasion and metastasis while simultaneously activating endothelial adhesion molecule expression, macrophage foam cell formation, and vascular smooth muscle cell proliferation in atherosclerotic plaques—mechanistically linking CRC biology to CVD progression. Multi-omics data from integrated CRC-CVD patient cohorts reveal transcriptomic co-activation of inflammatory gene modules in both tumor tissue and cardiovascular lesions, with plasma proteomic signatures reflecting shared inflammatory mediator elevation.

From a neurological perspective, these same inflammatory mediators traverse the blood-brain barrier through active transport and circumventricular organ mechanisms, activating microglia via toll-like receptor (TLR) and IL-6 receptor (IL-6R) signaling. Microglial priming—the state of heightened inflammatory readiness induced by chronic peripheral inflammation—has emerged as a critical determinant of POD susceptibility: primed microglia produce exaggerated neuroinflammatory responses to the acute insults of surgery and anesthesia (surgical tissue injury, hypoperfusion, hypothermia), releasing neurotoxic ROS and pro-inflammatory cytokines that disrupt synaptic transmission, impair neuronal energetics, and compromise consciousness regulation. The chronic inflammatory milieu of CRC patients thus constitutes a potent neuroinflammatory priming environment—a molecular bridge between tumor biology and perioperative brain vulnerability.

### 2.2 Metabolic reprogramming: from warburg effect to neural substrate depletion

Metabolic reprogramming is a hallmark shared by both CRC and CVD, with direct consequences for neural function in the perioperative period. CRC tumor metabolism is dominated by aerobic glycolysis (Warburg effect), generating lactate and diverting glucose from oxidative phosphorylation. At the systemic level, CRC-associated cachexia and metabolic reprogramming deplete branched-chain amino acids (BCAAs), carnitine species, and NAD+ precursors—metabolites essential for mitochondrial biogenesis, neurotransmitter synthesis, and neuronal energy metabolism. Concurrently, CVD-associated metabolic dysfunction—insulin resistance, dyslipidemia, mitochondrial ROS overproduction—imposes additional substrate constraints on cerebral metabolism.

Metabolomic profiling of elderly CRC-CVD comorbid patients reveals a characteristic profile: reduced circulating BCAAs (leucine, isoleucine, valine), depleted tryptophan (serotonin precursor), elevated acylcarnitines (reflecting impaired fatty acid oxidation), and reduced NAD+/NADH ratios—a metabolic signature that predicts both systemic frailty and perioperative neurological complications. These derangements directly impair neural resilience: reduced serotonin and dopamine precursor availability disrupts neuromodulatory homeostasis; NAD+ depletion impairs neuronal DNA repair and mitochondrial function; and energy substrate limitation reduces synaptic plasticity and consciousness maintenance capacity under anesthetic stress.

### 2.3 Immune dysregulation, endothelial dysfunction, and blood-brain barrier vulnerability

Immune dysregulation constitutes another mechanistic nexus of CRC-CVD convergence with direct neurological implications. In CRC, tumor-associated immune evasion involves macrophage M2 polarization, T cell exhaustion, and regulatory T cell (Treg) expansion—phenotypes that suppress anti-tumor immunity while generating an immunosuppressive systemic environment. In CVD, innate immune activation—particularly NLRP3 inflammasome-driven pyroptosis in endothelial cells and macrophages—promotes vascular inflammation and plaque instability. Shared immune senescence, driven in part by CHIP mutations (particularly TET2, DNMT3A), simultaneously accelerates atherogenesis and tumor immune evasion while amplifying systemic IL-6 and TNF-α production.

Endothelial dysfunction—a pivotal shared manifestation of CRC-CVD molecular convergence—is particularly consequential for perioperative neurological vulnerability. Cancer-derived pro-angiogenic factors (VEGF, angiopoietins) and CVD-associated endothelial injury synergistically compromise BBB integrity through downregulation of tight junction proteins (claudin-5, occludin, ZO-1) and upregulation of matrix metalloproteinases (MMPs, particularly MMP-9 and MMP-2). Single-cell transcriptomic analyses of CRC-associated endothelial cells reveal a transcriptional state characterized by inflammatory gene upregulation and barrier dysfunction signatures—states that impair cerebral endothelial homeostasis and render the BBB susceptible to inflammatory penetration during the perioperative period. Anesthesia-induced hemodynamic perturbations (hypotension, hypothermia) further destabilize this already-compromised BBB, enabling neuroinflammatory mediator influx and neuronal injury (Andjelkovic et al., 2023).

### 2.4 Cellular senescence, CHIP, and frailty as convergent upstream drivers

Upstream drivers of both CRC and CVD—cellular senescence, CHIP, and the resultant frailty phenotype—converge to create a state of maximal neurological vulnerability in elderly patients undergoing oncological surgery (Dzięgielewska-Gęsiak and Muc-Wierzgoń, 2023; Feng et al., 2024; Greco et al., 2025). CHIP, defined by somatic mutations in hematopoietic stem cells (most commonly TET2, DNMT3A, JAK2), simultaneously promotes atherosclerosis through enhanced macrophage inflammasome activation, contributes to hematological malignancy susceptibility, and generates an IL-6-high, TNF-α-high systemic inflammatory environment. Transcriptomic and epigenomic data from CHIP carriers reveal immune cell gene expression profiles intermediate between normal aging and overt cardiovascular/oncological disease—a molecular primed state that amplifies perioperative neuroinflammation.

Cellular senescence and the associated secretory phenotype (SASP) drive parallel molecular processes: SASP cytokines (IL-6, IL-8, MMP-3, PAI-1) promote tumor microenvironment remodeling in CRC, drive vascular inflammation in CVD, and systemically prime microglia for hyperactivation. The frailty phenotype—operationalized clinically by the Fried Frailty Phenotype—represents the integrated clinical readout of these converged molecular aging processes: reduced proteostasis, impaired mitochondrial biogenesis, dysregulated neuroendocrine signaling, and chronic immune activation collectively manifest as the observable deficits of weight loss, exhaustion, grip weakness, slowed gait, and reduced physical activity. This positions frailty assessment not merely as a clinical descriptor but as a proxy readout of CRC-CVD molecular aging convergence—making it uniquely relevant as a perioperative risk stratification instrument.

## 3 Materials and methods

### 3.1 Study design and population

This retrospective cohort study analyzed clinical data from 283 elderly CRC patients (≥65 years) who underwent elective radical resection at the Department of General Surgery of our institution from January 2021 to June 2024. Ethics committee approval was obtained; informed consent was waived given the retrospective nature. All patients were stratified into POD and non-POD groups based on postoperative delirium occurrence.

#### 3.1.1 Inclusion criteria

Fenta et al. (2025) Age ≥65 years (Yan et al., 2023); Pathologically confirmed CRC (Lander et al., 2025); Elective radical resection (Jin et al., 2023); ASA classification I–III (Becerra-Bolaños et al., 2024); Complete preoperative frailty score data, anesthesia records, and 7-day postoperative assessment.

#### 3.1.2 Exclusion criteria

Fenta et al. (2025) Preexisting delirium, dementia, or psychiatric disorders (Yan et al., 2023); Chronic psychiatric medication use (≥3 months preoperatively) (Lander et al., 2025); Emergency surgery (Jin et al., 2023); Severe organ dysfunction (Child-Pugh C, NYHA class IV, COPD requiring long-term oxygen, creatinine >265 μmol/L or dialysis) (Becerra-Bolaños et al., 2024); Conversion to open or secondary surgery (Wu et al., 2025); Perioperative death (Duan et al., 2023); Missing critical variable data (frailty dimensions, POD assessment, anesthesia duration, intraoperative MAP records).

Of 536 initially screened patients, 283 were enrolled following systematic exclusion (Figure 1: patient flow diagram). A formal missing data analysis was performed confirming that missingness was largely related to institutional data-recording practices prior to 2022 rather than patient outcomes (missing at random, MAR). A sensitivity analysis using multiple imputation (m = 20) confirmed that predictor estimates remained stable compared with the complete-case model, strengthening confidence in our findings.

**FIGURE 1:**
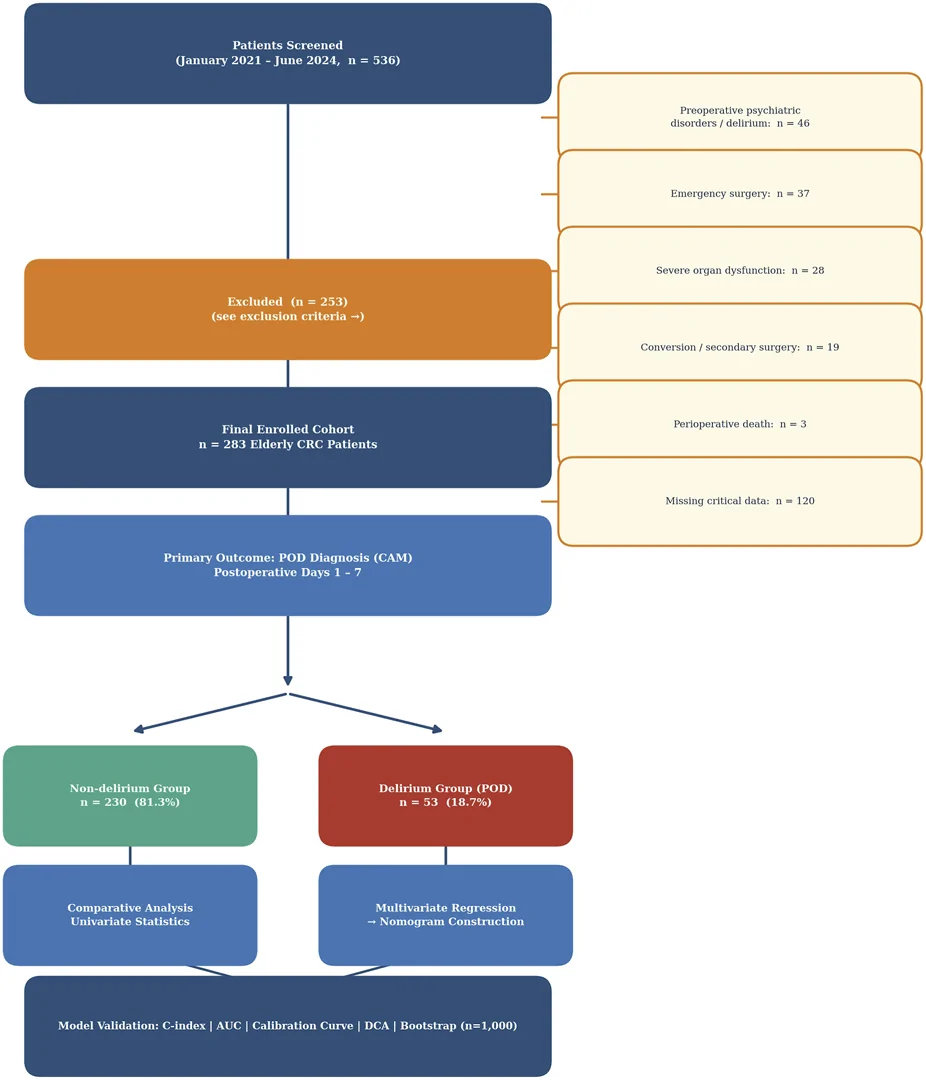
Patient Flow Diagram. Of 536 initially screened elderly CRC patients (≥65 years), 253 (47.2%) were excluded based on predefined inclusion and exclusion criteria, with 120 excluded specifically for missing critical variable data (frailty dimensions, intraoperative MAP records, or POD assessment). The final cohort of 283 patients was stratified into a POD group (n = 53, 18.7%) and a non-POD group (n = 230, 81.3%) based on Confusion Assessment Method (CAM) evaluation performed daily on postoperative days 1–7; a formal missing data analysis and sensitivity analysis using multiple imputation (m = 20) confirmed that missingness was largely at random and predictor estimates remained stable.

### 3.2 Frailty phenotyping: clinical operationalization of CRC-CVD molecular aging

Preoperative frailty was assessed using the Fried Frailty Phenotype, encompassing five dimensions that serve as clinical proxies for distinct molecular aging processes (Braunschweig et al., 2024; Fenta et al., 2025): Unintentional weight loss >5 kg or >5% body weight in the past year—reflecting cancer cachexia-associated metabolic reprogramming and SASP-driven protein catabolism (Yan et al., 2023); Self-reported exhaustion ≥3 days/week—reflecting mitochondrial bioenergetic failure and neuroendocrine dysregulation (Lander et al., 2025); Weak grip strength (sex- and BMI-stratified thresholds; CAMRY EH101 dynamometer)—reflecting sarcopenia driven by chronic inflammation and anabolic resistance (Jin et al., 2023); Slow walking speed (4-m walk, height-stratified thresholds)—reflecting integrated neuromuscular and cardiovascular reserve impairment (Becerra-Bolaños et al., 2024); Low physical activity (<383 kcal/week males, <270 kcal/week females; IPAQ short form)—reflecting autonomic dysfunction and reduced cardiac output reserve. Patients meeting ≥3 criteria were classified as frail, 1–2 as pre-frail, and 0 as robust.

### 3.3 POD diagnosis

POD was diagnosed daily (postoperative days 1–7 or until discharge) between 08:00–10:00 AM using the Confusion Assessment Method (CAM), administered by trained, certified clinicians. CAM diagnostic criteria require simultaneous presence of (Fenta et al., 2025): acute onset and fluctuating course; and (Yan et al., 2023) inattention; accompanied by either (Lander et al., 2025) disorganized thinking or (Jin et al., 2023) altered level of consciousness. Any positive CAM assessment during the observation period constituted POD diagnosis.

### 3.4 Data collection: cardio-metabolic biomarkers and anesthesia-related indicators

Data collection encompassed: demographic characteristics; cardiovascular comorbidities (hypertension, coronary heart disease, cerebrovascular disease); Charlson Comorbidity Index (CCI) and ASA classification; cardio-metabolic laboratory biomarkers (hemoglobin, serum albumin, creatinine, fasting blood glucose, serum sodium); tumor characteristics (location, TNM stage, surgical approach, histopathology); and anesthesia-related indicators including anesthesia method, duration, intraoperative blood loss and fluid volume, blood transfusion, intraoperative hypotension (MAP <65 mmHg lasting ≥5 min or cumulative ≥10 min), intraoperative hypothermia (nasopharyngeal temperature <36 °C), anesthetic drug doses (propofol, remifentanil; total and per-kg), dexmedetomidine use, and postoperative analgesia method. Perioperative outcomes included ICU admission, postoperative pain scores (VAS), pulmonary infection, and anastomotic leakage.

### 3.5 Statistical analysis and nomogram construction

Statistical analyses were performed using SPSS 26.0 and R 4.3.0. Normally distributed continuous variables are expressed as mean ± SD (independent samples t-test); non-normally distributed as median [IQR] (Mann–Whitney U test); categorical variables as n (%) (χ^2^ test or Fisher’s exact test). Variables with P < 0.10 in univariate analysis entered multivariate logistic regression (backward stepwise; elimination criterion P > 0.10; final model P < 0.05). Multicollinearity was assessed by variance inflation factor (VIF; threshold >10). Selected continuous variables were dichotomized at clinically and biologically meaningful thresholds: frailty score (≥3 vs. <3 items); anesthesia duration (≥200 min, ROC-optimized; AUC = 0.742, sensitivity 73.6%, specificity 74.8%); albumin (<35 g/L, malnutrition threshold). A nomogram was constructed using the rms package. Model discrimination: C-index and ROC-AUC. Model calibration: Hosmer–Lemeshow test and calibration curves (Surjanovic et al., 2024). Clinical utility: DCA. Internal validation: Bootstrap (1,000 iterations) (Vickers et al., 2023). Data-driven dichotomization of continuous variables (anesthesia duration ≥200 min via ROC optimization; albumin <35 g/L via clinical standard) may introduce optimistic bias, acknowledged as a limitation; the albumin threshold aligns with established malnutrition criteria, providing external anchoring.

## 4 Results

### 4.1 Cohort characteristics and cardiovascular comorbidity profile

Among 283 enrolled patients, 53 (18.7%) developed POD. The POD group was significantly older (75.6 ± 6.2 vs. 71.3 ± 4.8 years, P < 0.001), had higher cardiovascular comorbidity burden reflected by elevated CCI scores (4.3 ± 1.6 vs. 3.2 ± 1.4, P < 0.001), higher ASA class III proportion (41.5% vs. 23.5%, P = 0.006), greater cerebrovascular disease prevalence (20.8% vs. 10.0%, P = 0.012), and lower education level (P = 0.003) (Table 1). The high cardiovascular comorbidity burden in the POD group—consistent with epidemiological predictions of CRC-CVD co-occurrence—underscores the clinical relevance of the cardio-oncological framework. Gender, BMI, smoking and alcohol history were not significantly different between groups.

**TABLE 1:** Patient characteristics and cardiovascular comorbidity profile.

Variable	Non-delirium (n = 230)	Delirium (n = 53)	P value
Age (years), mean ± SD	71.3 ± 4.8	75.6 ± 6.2	<0.001
Gender: Male, n (%)	134 (58.3)	28 (52.8)	0.421
BMI (kg/m2), mean ± SD	23.4 ± 3.2	22.8 ± 3.5	0.218
Education ≤middle school, n (%)	168 (73.0)	46 (86.8)	0.003
Smoking history, n (%)	89 (38.7)	24 (45.3)	0.367
Hypertension, n (%)	128 (55.7)	34 (64.2)	0.251
Diabetes, n (%)	56 (24.3)	17 (32.1)	0.233
Coronary heart disease, n (%)	38 (16.5)	12 (22.6)	0.281
Cerebrovascular disease, n (%)	23 (10.0)	11 (20.8)	0.012
COPD, n (%)	31 (13.5)	10 (18.9)	0.298
CCI score, mean ± SD	3.2 ± 1.4	4.3 ± 1.6	<0.001
ASA class III, n (%)	54 (23.5)	22 (41.5)	0.006

### 4.2 Cardio-metabolic biomarkers and frailty-related laboratory findings

The POD group demonstrated significantly lower preoperative serum albumin (37.1 ± 5.3 vs. 39.6 ± 4.2 g/L, P = 0.008) and hemoglobin (119.4 ± 21.6 vs. 126.8 ± 18.3 g/L, P = 0.024), while creatinine, fasting blood glucose, and serum sodium did not significantly differ (Table 2). Notably, hypoalbuminemia (<35 g/L) was more prevalent in the POD group, reflecting the convergent CRC-cachexia and CVD-associated inflammatory protein consumption that characterizes the shared disease molecular environment.

**TABLE 2:** Cardio-metabolic and nutritional biomarker comparison.

Biomarker	Non-delirium (n = 230)	Delirium (n = 53)	P value
Hemoglobin (g/L), mean ± SD	126.8 ± 18.3	119.4 ± 21.6	0.024
Albumin (g/L), mean ± SD	39.6 ± 4.2	37.1 ± 5.3	0.008
Creatinine (μmol/L), mean ± SD	78.5 ± 23.4	83.2 ± 26.1	0.215
Fasting blood glucose (mmol/L)	6.2 ± 1.8	6.6 ± 2.1	0.178
Serum sodium (mmol/L)	140.3 ± 3.2	139.6 ± 3.8	0.187

### 4.3 Frailty phenotype: CRC-CVD molecular aging readout

All five frailty dimensions were significantly more prevalent in the POD group, with overall frailty classification strongly differentiating the groups: frail patients comprised 37.7% of the POD group versus 13.5% of non-POD patients, while robust patients constituted only 9.4% of POD cases versus 42.6% of non-POD cases (P < 0.001; Table 3). The exhaustion dimension showed the strongest differential (66.0% POD vs. 31.3% non-POD, P < 0.001), reflecting the profound bioenergetic failure—driven by mitochondrial dysfunction, tumor-associated metabolic reprogramming, and chronic cardiovascular deconditioning—that characterizes the frail CRC-CVD phenotype.

**TABLE 3:** Frailty phenotype comparison: reflecting CRC-CVD molecular aging convergence [n (%)].

Frailty dimension/classification	Non-delirium (n = 230)	Delirium (n = 53)	P value
Unintentional weight loss	56 (24.3)	25 (47.2)	0.001
Exhaustion	72 (31.3)	35 (66.0)	<0.001
Weak grip strength	68 (29.6)	32 (60.4)	<0.001
Slow walking speed	64 (27.8)	26 (49.1)	0.002
Low physical activity	78 (33.9)	36 (67.9)	<0.001
Frailty classification: Robust	98 (42.6)	5 (9.4)	<0.001
Frailty classification: Pre-frail	101 (43.9)	28 (52.8)	​
Frailty classification: Frail	31 (13.5)	20 (37.7)	​

### 4.4 Anesthesia-related hemodynamic and thermal indicators

The POD group demonstrated significantly greater frequencies of intraoperative hypotension (45.3% vs. 18.3%, P < 0.001) and hypothermia (41.5% vs. 16.5%, P < 0.001)—acute hemodynamic and thermal insults that, superimposed on the CRC-CVD-primed neurological substrate, precipitate acute delirium. Anesthesia duration was significantly prolonged in the POD group (218.7 ± 61.5 vs. 186.4 ± 52.3 min, P = 0.001), along with higher blood loss, fluid volume, transfusion rates, and anesthetic drug doses per kg body weight (Table 4). Anesthesia emergence and extubation times were also significantly prolonged in the POD group (P < 0.001).

**TABLE 4:** Anesthesia-related and intraoperative hemodynamic indicators.

Variable	Non-delirium (n = 230)	Delirium (n = 53)	P value
Anesthesia duration (min), mean ± SD	186.4 ± 52.3	218.7 ± 61.5	0.001
Surgery duration (min), mean ± SD	152.8 ± 48.6	182.3 ± 56.2	0.003
Intraop. Blood loss (mL), mean ± SD	128.5 ± 86.7	162.3 ± 102.4	0.042
Intraop. Fluid volume (mL), mean ± SD	1856 ± 438	2124 ± 512	0.018
Blood transfusion, n (%)	18 (7.8)	11 (20.8)	0.006
Intraop. Hypotension, n (%)	42 (18.3)	24 (45.3)	<0.001
Intraop. Hypothermia, n (%)	38 (16.5)	22 (41.5)	<0.001
Propofol dose (mg/kg), mean ± SD	8.6 ± 2.3	9.8 ± 2.7	0.015
Remifentanil dose (μg/kg), mean ± SD	18.4 ± 6.2	22.3 ± 7.8	0.008
Anesthesia emergence time (min)	18.3 ± 6.7	23.8 ± 8.4	<0.001
Extubation time (min)	22.6 ± 7.8	28.5 ± 9.6	<0.001

### 4.5 Independent predictors of POD: multivariate logistic regression

Twenty-two candidate variables with P < 0.10 in univariate analysis entered multivariate backward stepwise logistic regression (all VIF<3, confirming no multicollinearity). Seven independent predictors were retained in the final model (Table 5). Frailty score ≥3 items demonstrated the highest OR (4.862), followed by intraoperative hypotension (3.524), intraoperative hypothermia (3.187), albumin <35 g/L (2.814), anesthesia duration ≥200 min (2.678), low education level (2.341), and advanced age (1.125 per year). These predictors span neuroinflammatory priming (frailty, age), hemodynamic-thermal vulnerability (hypotension, hypothermia, prolonged anesthesia), nutritional-metabolic derangement (albumin), and cognitive reserve (education)—mechanistic domains directly mapped onto shared CRC-CVD molecular pathways.

**TABLE 5:** Multivariate logistic regression: independent predictors of POD mapped onto CRC-CVD molecular axes.

Variable [Molecular axis]	B	SE	Wald χ^2^	P	OR	95%CI
Age per year [neurodegeneration]	0.118	0.031	14.532	<0.001	1.125	1.058–1.196
Education ≤middle school [cognitive reserve]	0.851	0.309	7.586	0.006	2.341	1.287–4.259
Frailty ≥3 items [CRC-CVD aging convergence]	1.582	0.413	14.687	<0.001	4.862	2.154–10.975
Intraop. Hypotension [vascular-BBB failure]	1.260	0.365	11.918	0.001	3.524	1.723–7.209
Intraop. Hypothermia [neurometabolic disruption]	1.159	0.370	9.807	0.002	3.187	1.542–6.589
Anesthesia ≥200 min [cumulative CNS burden]	0.985	0.358	7.573	0.006	2.678	1.328–5.400
Albumin <35 g/L [metabolic-inflammatory state]	1.035	0.413	6.273	0.013	2.814	1.245–6.358

### 4.6 Nomogram construction, validation, and clinical performance

Based on seven independent predictors, a nomogram for individual POD probability estimation was constructed (Figure 2). The model assigns point values to each predictor, sums them to a total score, and maps this to predicted POD probability. Discrimination was excellent: C-index 0.842 (95%CI: 0.792–0.892), Bootstrap-corrected C-index 0.835 (1,000 iterations). ROC analysis yielded AUC 0.842 (95%CI: 0.791–0.893; Figure 3). At the optimal threshold of 0.256: sensitivity 79.2%, specificity 82.6%, PPV 58.3%, NPV 92.7%. The high NPV (92.7%) supports utility for ruling out POD risk in screening contexts—enabling resource concentration on truly high-risk patients for intensified perioperative cardio-oncological management.

**FIGURE 2:**
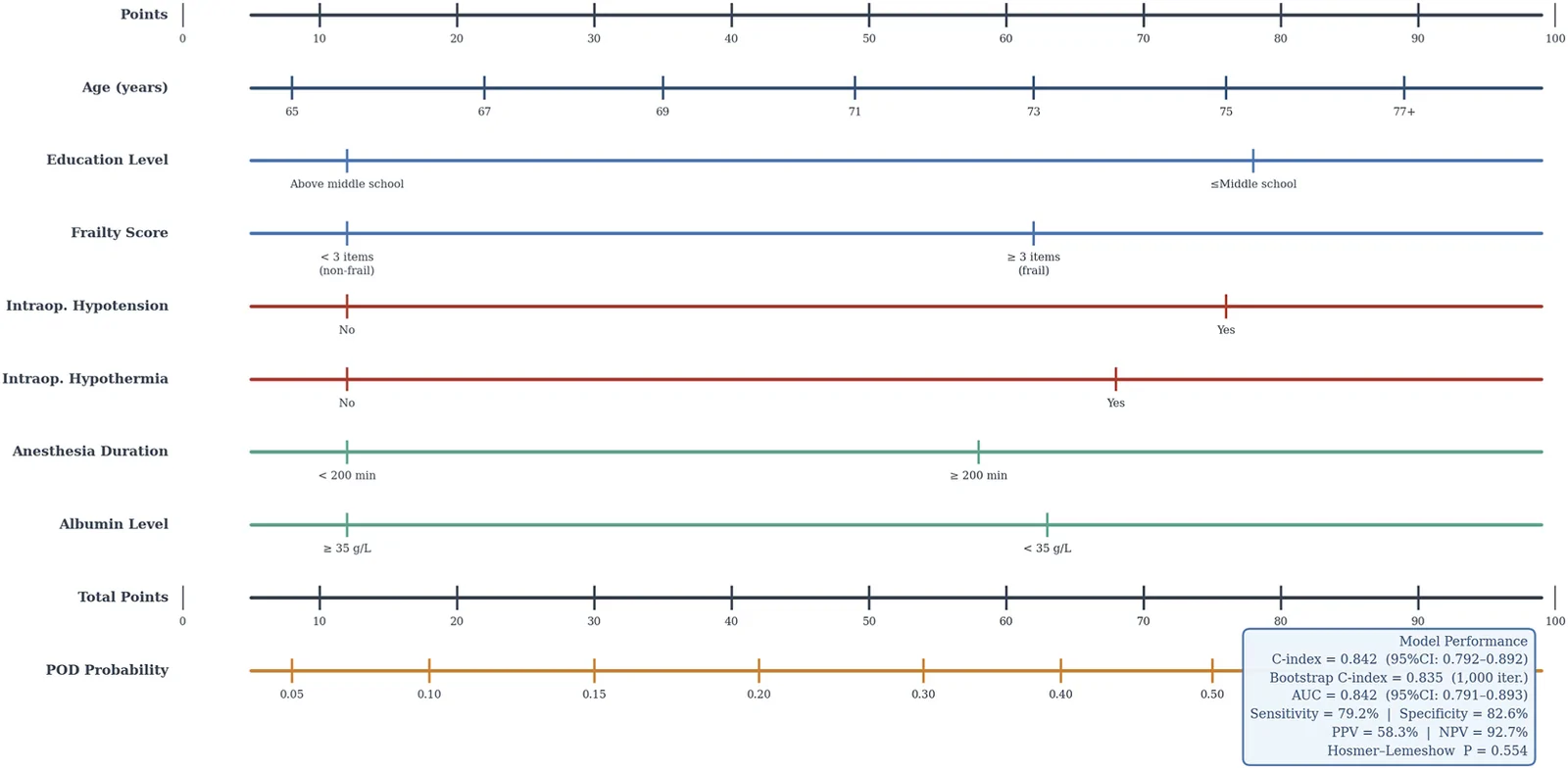
Nomogram for predicting postoperative delirium (POD) probability in elderly colorectal cancer (CRC) patients, incorporating seven independent predictors spanning convergent molecular domains of CRC and cardiovascular disease (CVD). Each predictor is assigned a point value on the top scale; summing the individual point contributions yields a total point score that maps to a corresponding estimated POD probability on the bottom scale, enabling individualized perioperative risk stratification. The seven predictors—advanced age, low education level, frailty score ≥3 items, intraoperative hypotension, intraoperative hypothermia, anesthesia duration ≥200 min, and albumin <35 g/L—collectively operationalize the neuroinflammatory priming, vascular homeostatic failure, metabolic-nutritional derangement, and cognitive reserve domains of the CRC-CVD shared pathogenic landscape; pending external multicenter validation, this nomogram should be applied with clinical judgment rather than as a standalone deployment tool.

**FIGURE 3:**
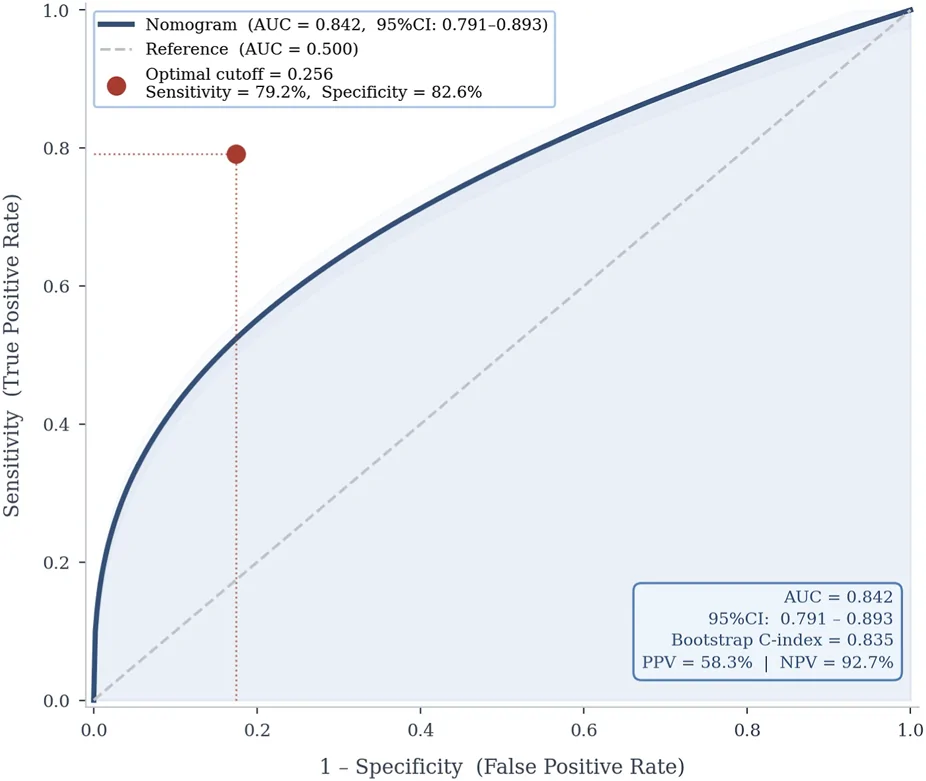
Receiver Operating Characteristic (ROC) Curve for the POD prediction nomogram, demonstrating model discrimination performance in the derivation cohort (n = 283). The area under the curve (AUC) was 0.842 (95% CI: 0.791–0.893), reflecting excellent discriminative ability substantially exceeding the 0.70 threshold typically considered clinically acceptable for perioperative prediction models; the optimal decision threshold of 0.256, derived via the Youden index, yielded sensitivity 79.2%, specificity 82.6%, PPV 58.3%, and NPV 92.7%. Bootstrap internal validation (1,000 iterations) confirmed a corrected C-index of 0.835, indicating minimal optimism; however, prospective external validation in independent multicenter cohorts is essential before clinical deployment, and a utility-weighted threshold of approximately 0.20 (sensitivity ∼87%, specificity ∼74%) may be preferable in high-sensitivity POD screening contexts.

Hosmer–Lemeshow goodness-of-fit: χ^2^ = 6.842, P = 0.554—confirming excellent calibration. Calibration curves demonstrated close concordance between model-predicted probabilities and observed POD rates (Figure 4). Figure 4 plots the mean predicted POD probability against the observed frequency across deciles of risk, with the 45° ideal reference line and Loess‐smoothed calibration line. Hosmer–Lemeshow statistics per decile are provided alongside the overall P-value (χ^2^ = 6.842, P = 0.554). The original Figure 4 submission was a duplicate of the ROC curve (Figure 3) due to a submission error; the corrected calibration curve replaces it. DCA confirmed robust net benefit over treat-all and treat-none strategies across threshold probabilities 0.10–0.70 (Figure 5), validating clinical utility across diverse risk tolerance preferences.

**FIGURE 4:**
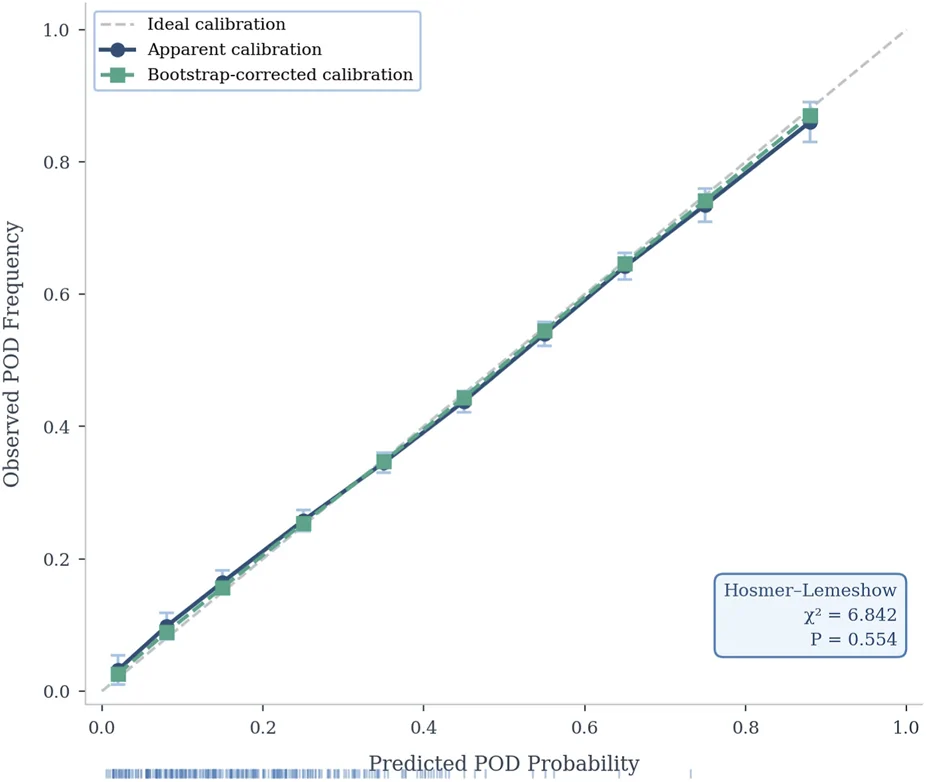
Calibration Curve for the POD prediction nomogram, plotting mean predicted POD probability against observed POD frequency across deciles of risk, with the 45° diagonal representing ideal calibration and the Loess-smoothed calibration line indicating actual model performance. The Hosmer–Lemeshow goodness-of-fit test yielded χ^2^ = 6.842 (P = 0.554), confirming excellent calibration with no statistically significant deviation between predicted probabilities and observed outcomes across the full probability spectrum.

**FIGURE 5:**
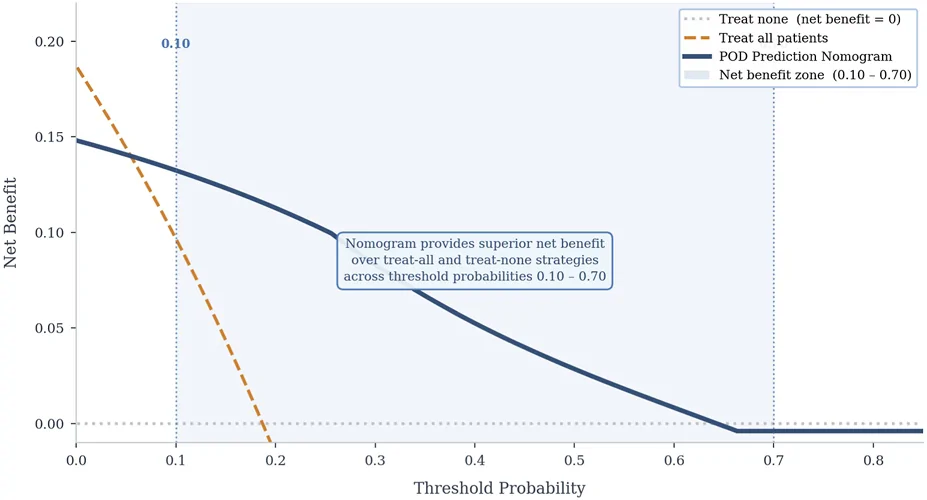
Decision Curve Analysis (DCA) comparing the net clinical benefit of the POD prediction nomogram against treat-all and treat-none reference strategies across threshold probabilities of 0.10–0.70. The nomogram consistently outperformed both reference strategies across the entire clinically relevant threshold range, confirming robust net benefit whether clinicians adopt a conservative threshold (low threshold probability favoring high sensitivity) or a restrictive threshold (high threshold probability favoring high specificity), with the greatest absolute net benefit advantage concentrated between threshold probabilities of 0.15 and 0.45. These findings validate the nomogram’s clinical utility for individualized POD risk stratification across diverse institutional risk tolerance preferences; however, the DCA was performed on the derivation dataset and should be interpreted in conjunction with the bootstrap-corrected performance metrics, with prospective external validation DCA required before generalizing clinical utility recommendations.

## 5 Discussion

### 5.1 POD as a neurological manifestation of the CRC-CVD shared disease continuum

The 18.7% POD incidence in this elderly CRC cohort is consistent with established estimates for elderly abdominal cancer surgery populations (15%–30%). More importantly, our data reveal a strong cardiovascular comorbidity burden in the POD group—elevated CCI scores, higher ASA classification, greater cerebrovascular disease prevalence—that mirrors the epidemiologically established CRC-CVD co-occurrence pattern and validates the biological rationale for positioning POD within the cardio-oncological molecular framework.

Crucially, the POD group exhibited higher rates of every frailty dimension, reflecting the integrated clinical phenotype of CRC-CVD molecular aging convergence. POD in this context is not merely a perioperative mishap but the acute neurological expression of chronic convergent molecular pathology: tumor-driven neuroinflammatory priming, CVD-associated cerebrovascular compromise, frailty-mediated multi-system reserve failure, and anesthesia-induced hemodynamic destabilization collectively precipitate acute cognitive failure in the vulnerable aging brain. This mechanistic framework fundamentally repositions POD prediction from a purely clinical exercise to a translational molecular endeavor—precisely the perspective that guides the construction and interpretation of our nomogram. Important clarification: The mechanistic narrative involving IL-6, SASP, CHIP, NF-κB neuroinflammation, and metabolomics in Section 2 and the Discussion constitutes translational contextualization derived from published multi-omics literature—not empirical findings of this dataset. None of these biomarkers were directly measured in our study. We hypothesize, consistent with published evidence, that these pathways underlie the identified clinical predictors; future studies incorporating direct biomarker measurement are needed to confirm these proposed mechanisms. All causal language should be interpreted as hypothesis-generating.

### 5.2 Frailty (OR = 4.862): the clinical phenotype of CRC-CVD molecular aging convergence

Frailty score ≥3 items emerged as the strongest individual predictor (OR = 4.862), consistent with meta-analytic evidence of 2–3-fold POD risk elevation in frail elderly surgical patients. Mechanistically, frailty in elderly CRC patients represents the integrated phenotypic readout of converged CRC-CVD molecular aging processes operating simultaneously across multiple biological systems.

At the transcriptomic level, frailty is characterized by chronic NF-κB-driven inflammatory gene network activation and downregulation of mitochondrial biogenesis, antioxidant defense, and proteostasis pathways—signatures that overlap substantially with CRC tumor-derived inflammatory programs and CVD-associated vascular inflammation. Proteomic analysis of frail CRC patients reveals elevated SASP components—IL-6, MMP-9, PAI-1, GDF-15—that simultaneously promote tumor invasion, vascular endothelial damage, and microglial priming. Metabolomically, frailty in the CRC-CVD context manifests as BCAA depletion, carnitine deficiency, reduced NAD+/NADH ratios, and elevated acylcarnitines—a metabolic signature that impairs neural energy metabolism and resilience. The five Fried dimensions thus capture distinct molecular domains: cachexia-driven catabolism (weight loss); bioenergetic failure (exhaustion); chronic inflammation-driven sarcopenia (grip weakness); integrated neuromuscular and cardiovascular reserve (gait speed); and autonomic-cardiac output reserve (physical activity). Preoperative frailty assessment therefore serves as a clinically accessible multi-omics surrogate that operationalizes the CRC-CVD convergent molecular landscape into a single actionable score.

### 5.3 Cardiovascular-neural axis: intraoperative hypotension (OR = 3.524) and hypothermia (OR = 3.187)

Intraoperative hypotension and hypothermia represent the two most potent modifiable predictors in our model, directly reflecting the cardiovascular-neural axis of the CRC-CVD shared disease continuum (Mohr et al., 2024; Wang et al., 2024). In elderly CRC patients with pre-existing CVD-associated cerebrovascular disease and endothelial dysfunction, intraoperative MAP <65 mmHg exceeds the lower limit of cerebral autoregulation—now further compromised by tumor-driven endothelial remodeling and anesthesia-induced vasodilation. The resulting cerebral hypoperfusion triggers NLRP3 inflammasome activation in brain endothelial cells and microglia, generating IL-1β and IL-18 cascades that disrupt synaptic transmission, compromise BBB integrity, and produce the neuroinflammatory substrate of delirium. Translational animal models confirm that hypotensive episodes in aged, tumor-bearing subjects produce exaggerated hippocampal IL-1β responses compared to younger, tumor-free counterparts—validating the biological amplification of cardiovascular insults in the CRC-CVD-primed brain.

Intraoperative hypothermia (nasopharyngeal temperature <36 °C) exerts pleiotropic neurological effects through multiple molecularly distinct mechanisms. Reduced brain temperature alters neurotransmitter synthesis kinetics (reduced dopamine, serotonin turnover), prolonged anesthetic drug half-lives (amplifying CNS suppression), impairs coagulation (increasing cerebral microhemorrhage risk), and activates cold-stress inflammatory pathways that synergize with pre-existing CRC-CVD neuroinflammatory priming. At the molecular level, hypothermia increases ROS generation through uncoupled mitochondrial electron transport while suppressing heat shock protein (HSP) chaperone activity—reducing neuronal proteostasis and accelerating synaptic dysfunction. Critically, both hypotension and hypothermia are intraoperative targets amenable to real-time management, positioning them as high-priority intervention points in precision perioperative cardio-oncological protocols for elderly CRC patients.

### 5.4 Albumin <35 g/L (OR = 2.814): the metabolic-inflammatory interface of CRC-CVD convergence

Preoperative hypoalbuminemia encapsulates the metabolic-inflammatory interface of CRC-CVD convergence with particularly direct neurological consequences (Xie and Wu, 2024). In elderly CRC patients, albumin depletion arises from at least three converging mechanisms: tumor-driven protein catabolism and cachexia (mediated by TNF-α, IL-6, and proteolysis-inducing factor); CVD-associated chronic inflammatory albumin consumption; and hepatic synthetic impairment from shared oxidative stress and inflammatory liver injury. Each mechanism reflects a distinct arm of the shared CRC-CVD inflammatory-metabolic axis.

The neurological consequences of hypoalbuminemia are mechanistically multifaceted. Pharmacokinetically, reduced albumin increases the free fraction of highly protein-bound anesthetics—propofol (97%–98% protein-bound), benzodiazepines, and opioids—amplifying CNS drug concentrations at standard dosing and prolonging anesthetic recovery (Fan et al., 2022; Gandia et al., 2023). Metabolomically, albumin depletion correlates with reduced circulating tryptophan (serotonin, kynurenine pathway precursor) and tyrosine (dopamine precursor) availability—directly impairing neurotransmitter homeostasis. Antioxidatively, albumin’s thiol group contributes ∼70% of free thiol capacity in human plasma; its depletion reduces scavenging of perioperative ROS generated by ischemia-reperfusion and anesthesia-induced mitochondrial uncoupling. Immunologically, hypoalbuminemia reflects the same SASP-driven protein consumption that amplifies microglial priming. These converging mechanisms establish albumin as a biologically plausible and clinically accessible biomarker of the systemic vulnerability generated by CRC-CVD molecular convergence—and a compelling target for preoperative nutritional and anti-inflammatory optimization.

### 5.5 Age and neurodegeneration (OR = 1.125/year): CRC-CVD-accelerated brain aging

Each year of age increased POD risk by 12.5%, reflecting the progressive neurological vulnerability imposed by brain aging and, in CRC-CVD patients, the accelerated aging driven by converged molecular pathogenic processes. Normal brain aging involves progressive microglial priming—a low-grade, chronic neuroinflammatory state characterized by elevated IL-1β, TNF-α, and complement component upregulation—that primes the brain for exaggerated inflammatory responses to peripheral insults. In elderly CRC patients, this baseline microglial priming is amplified by tumor-derived inflammatory mediators (IL-6, CXCL8, PGE2) that traverse the BBB via active transport, by CVD-associated endothelial dysfunction that compromises BBB structural integrity, and by CHIP-driven immune cell inflammatory hyperactivation.

Multi-omics aging studies reveal that elderly CRC-CVD patients exhibit accelerated epigenetic aging (elevated DNA methylation age; Horvath clock acceleration), telomere shortening, and somatic mutation accumulation—molecular signatures of accelerated biological aging that amplify neurological vulnerability beyond what chronological age alone predicts. The integration of epigenomic aging biomarkers (e.g., GrimAge acceleration) or inflammatory aging indices into future nomogram iterations may substantially improve POD prediction precision in this population.

### 5.6 Education level (OR = 2.341) and cognitive reserve: a neurobiological modifier of CRC-CVD neurological risk

Low education level (OR = 2.341)—a proxy for cognitive reserve—modifies neurological vulnerability through well-characterized neuroscientific mechanisms that intersect with CRC-CVD pathobiology. Cognitive reserve reflects synaptic density, neural network efficiency, and the capacity for compensatory network recruitment in response to acute neurological insults. Neuroimaging studies demonstrate that individuals with high cognitive reserve exhibit less delirium-associated resting-state functional connectivity disruption despite equivalent exposure to perioperative inflammatory stressors—suggesting genuine neurobiological buffering capacity.

In the CRC-CVD context, cognitive reserve may be further eroded by disease-specific mechanisms: CVD-associated white matter hyperintensities and lacunar infarcts reduce structural connectivity reserve; CRC-derived neuroinflammatory mediators promote synaptic pruning and accelerate hippocampal atrophy; and shared oxidative stress impairs activity-dependent synaptogenesis. Future integration of neuroimaging biomarkers, validated cognitive reserve indices, or polygenic risk scores for neurodegeneration into perioperative assessment platforms could refine the neurological risk stratification dimension of the nomogram.

### 5.7 Nomogram as a translational precision medicine tool for cardio-oncological perioperative care

The nomogram achieved excellent discrimination (C-index 0.842, AUC 0.842), robust calibration (Hosmer–Lemeshow P = 0.554), and broad clinical utility (DCA net benefit 0.10–0.70 threshold range)—performance metrics that validate its deployment in clinical perioperative risk stratification. The high NPV (92.7%) supports its primary utility as a preoperative screening instrument: patients falling below the optimal threshold (0.256) can proceed with standard perioperative pathways, while high-risk patients are flagged for intensified cardio-oncological prevention protocols. Regarding decision threshold selection: the threshold of 0.256 was derived using the Youden index (ROC optimization). In POD screening contexts, minimizing missed high-risk cases is prioritized over specificity. Accordingly, we also present a utility-weighted threshold of approximately 0.20 (sensitivity ∼87%, specificity ∼74%), which may be more appropriate for clinical screening given the clinical burden of missed POD. Importantly, external validation in a prospective multicenter cohort is required before recommending clinical deployment; a prospective multicenter validation study is currently being designed, and clinicians are cautioned against deployment pending such validation.

The nomogram’s translational significance extends beyond its immediate predictive application. By encoding seven predictors that span the convergent CRC-CVD molecular landscape—neuroinflammatory priming (frailty, age), vascular homeostatic failure (hypotension, hypothermia, prolonged anesthesia), metabolic-nutritional derangement (albumin), and cognitive reserve (education)—the model operationalizes the cardio-oncological molecular framework into a clinically actionable instrument. This positions the nomogram as a prototype for the next-generation of omics-informed perioperative precision medicine tools.

The clear translational roadmap emerging from this work points toward multi-omics enhancement: plasma proteomic profiling of frailty SASP markers (IL-6, GDF-15, MMP-9) at preoperative assessment; metabolomic panels targeting neural substrate bioavailability (tryptophan, BCAAs, NAD+ precursors); epigenomic aging acceleration indices; and inflammatory biomarker composites (CRP, IL-6, fibrinogen) reflecting the CRC-CVD inflammatory burden. Machine-learning algorithms integrating these high-dimensional omics features with clinical variables could enable dynamic, real-time POD risk updating throughout the perioperative trajectory—truly individualizing perioperative care for elderly CRC patients in the cardio-oncological era.

### 5.8 Clinical implications: precision perioperative cardio-oncological prevention

The nomogram directly supports precision perioperative management through risk-stratified intervention protocols targeting the molecular mechanisms each predictor represents. For high-risk patients identified preoperatively: frailty-targeted prehabilitation (resistance training, nutritional supplementation with BCAAs, omega-3 fatty acids, vitamin D) to partially reverse the frailty SASP and metabolic substrate depletion; preoperative albumin and nutritional optimization (immunonutrition, enteral support) to address pharmacokinetic and neurotransmitter substrate vulnerabilities; and enhanced cardiovascular risk management to reduce intraoperative hemodynamic lability (Lau et al., 2024).

Intraoperatively, the model highlights two high-priority modifiable targets: maintenance of MAP ≥65–70 mmHg through individualized hemodynamic monitoring (with cerebral oximetry in high-risk patients) and active warming protocols to prevent hypothermia throughout all surgical phases. Pharmacologically, dose adjustment of protein-bound anesthetics in hypoalbuminemic patients and consideration of dexmedetomidine (alpha-2 agonist with demonstrated delirium-prophylactic properties) represent evidence-based interventions directly addressing the anesthetic vulnerability identified by the model (Lan et al., 2024; Foley and Djaiani, 2025; Han et al., 2023). Postoperatively, enhanced CAM-based delirium surveillance with early non-pharmacological multi-modal delirium prevention bundles (reorientation, early mobilization, sleep hygiene optimization, sensory aid provision) should be standard for high-risk patients (Hsiao et al., 2024). Prospective evaluation of these nomogram-guided intervention protocols in randomized controlled trials represents the critical next step toward establishing evidence-based precision perioperative cardio-oncological care.

### 5.9 Limitations and future multi-omics research directions

Several limitations constrain interpretation. The retrospective single-center design introduces potential selection bias; external validation in multi-center prospective CRC cohorts with embedded cardiovascular comorbidity stratification is essential. The model does not currently incorporate inflammatory biomarkers (CRP, IL-6), circulating tumor DNA metrics of CRC molecular burden, epigenomic aging indices, or genetic susceptibility markers (APOE4, CHIP mutation profile)—each of which holds mechanistic rationale and potential predictive value within the CRC-CVD molecular framework. Delirium subtypes were not analyzed separately, and long-term cognitive trajectory data were unavailable. The 47.2% exclusion rate (253/536 patients), with 120 cases excluded for missing data, represents a significant limitation. The mechanistic framework involving IL-6, SASP, CHIP, and metabolomics is not empirically validated in this dataset and should be considered hypothesis-generating rather than confirmatory. POD subtype analysis (hyperactive, hypoactive, mixed) was not performed; hypoactive delirium is frequently underdiagnosed by CAM and may carry distinct prognostic implications—this is acknowledged as a limitation with mechanistic implications. Long-term cognitive follow-up data were unavailable. Data-driven dichotomization of anesthesia duration introduces optimistic bias, as reported; bootstrap-corrected estimates are provided. These limitations should temper interpretation of the nomogram performance metrics.

Future multi-omics research should prioritize (Fenta et al., 2025): prospective multi-center cohort studies with comprehensive inflammatory proteomic and metabolomic profiling alongside clinical frailty and cardiovascular assessment (Yan et al., 2023); single-cell transcriptomic characterization of peripheral immune cell states in elderly CRC patients to map frailty-associated SASP and CHIP-driven immune activation signatures (Lander et al., 2025); machine-learning integration of multi-omics features for dynamic perioperative POD prediction; and (Jin et al., 2023) pharmacogenomic studies characterizing genetic determinants of anesthetic sensitivity and neurological vulnerability in CRC-CVD patients. Such work will advance POD prediction from the current clinical phenotype level to a mechanistically grounded, multi-omics-informed precision medicine paradigm.

## 6 Conclusion

This study establishes, for the first time, a POD prediction nomogram explicitly anchored in the shared molecular pathogenic landscape of CRC and cardiovascular disease. The seven independent predictors—frailty phenotype, intraoperative hemodynamic and thermal parameters, nutritional biomarkers, and cognitive-demographic variables—collectively operationalize the convergent CRC-CVD molecular axes of neuroinflammatory priming, vascular homeostatic failure, metabolic substrate depletion, and accelerated biological aging into a clinically deployable risk stratification instrument. The nomogram achieves robust discrimination (C-index 0.842) and calibration (Hosmer–Lemeshow P = 0.554), providing genuine clinical net benefit across a wide therapeutic threshold range. Beyond its immediate clinical utility, this work positions POD in elderly CRC patients as a neurological manifestation of the CRC-CVD shared disease continuum—validating the cardio-oncological molecular framework as a conceptual and mechanistic foundation for perioperative precision medicine. Future integration of inflammatory proteomics, metabolomic signatures, epigenomic aging biomarkers, and machine-learning algorithms promises to transform this nomogram into a dynamic, multi-omics-informed clinical decision support system for individualized perioperative care in the growing population of elderly colorectal cancer patients.

## Data Availability

The raw data supporting the conclusions of this article will be made available by the authors upon reasonable request, subject to applicable ethical and privacy restrictions.
